# Antigenic variations of recent street rabies virus

**DOI:** 10.1080/22221751.2019.1683436

**Published:** 2019-11-04

**Authors:** Wenbo Wang, Jian Ma, Jianhui Nie, Jia Li, Shouchun Cao, Lan Wang, Chuanfei Yu, Weijin Huang, Yuhua Li, Yongxin Yu, Mifang Liang, Brett Zirkle, Xiaojiang S. Chen, Xuguang Li, Wei Kong, Youchun Wang

**Affiliations:** a College of Life Science, Jilin University, Changchun, People’s Republic of China; b Division of Monoclonal Antibody Products, National Institutes for Food and Drug Control (NIFDC), Beijing, People’s Republic of China; c Division of HIV/AIDS and Sex-Transmitted Virus Vaccines, National Institutes for Food and Drug Control (NIFDC), Beijing, People’s Republic of China; d Division of Arboviral Vaccine, National Institutes for Food and Drug Control (NIFDC), Beijing, People’s Republic of China; e Key Laboratory for Medical Virology, NHFPC, National Institute for Viral Disease Control and Prevention, China CDC, Beijing, People’s Republic of China; f Department of Molecular and Computational Biology/Chemistry Department/Norris Cancer Center, University of Southern California, Los Angeles, CA, USA; g Centre for Biologics Evaluation, Biologics and Genetic Therapies Directorate, Health Canada, Canada

**Keywords:** Rabies virus, glycoprotein, vaccine, monoclonal antibody, neutralization

## Abstract

The genetic and/or antigenic differences between street rabies virus (RABV) and vaccine strains could potentially affect effectiveness of rabies vaccines. As such, it is important to continue monitoring the glycoprotein (G) of the street isolates. All RABVG sequences in public database were retrieved and analysed. Using a pseudovirus system, we investigated 99 naturally occurring mutants for their reactivities to well-characterized neutralizing monoclonal antibodies (mAbs) and vaccine-induced antisera. A divergence in G sequences was found between vaccine strains and recent street isolates, with mutants demonstrating resistance to neutralizing mAbs and vaccine-induced antibodies. Moreover, antigenic variants were observed in a wide range of animal hosts and geographic locations, with most of them emerging since 2010. As the number of antigenic variants has increased in recent years, close monitoring on street isolates should be strengthened.

## Introduction

Rabies is caused by Rabies virus (RABV), a lyssavirus in the family *Rhabdoviridae*. Once clinical signs and symptoms develop, nearly all patients succumb to the fatal infection [1]. Vaccination is the most effective means to prevent rabies. The vaccine strains for humans are derived from various viral strains including the Pasteur virus strain (PV) and its attenuated forms in Pitman-Moore(PM) and PV-2061, the low egg passage strain Flury-LEP isolated in 1939, the Vnukovo-32 strain [2] isolated in North America in 1935, and two Chinese strains (CTN-1V and aGV) isolated in 1953 and 1931, respectively.

The effectiveness of vaccines could potentially be affected by genetic and/or antigenic differences between virus and vaccine strains [3–5]. Note that effectiveness of the rabies vaccines may vary depending on the viruses; specifically, the vaccines generally protect the hosts against phylogroup I lyssaviruses [6–9], while little or no protection against more genetic and antigenic divergent phylogroup II & III lyssaviruses [8,10,11]. While the current rabies vaccines have an excellent tracking record affording effective protection against RABVs tested to date [12], potential risk remains if street RABVs evolve resulting in significantly altered antigenicity. Indeed, the G gene sequences of street RABV isolates were reported to different from the current vaccine strains in some regions [13–15], while numerous street viruses have been shown to be antigenically different from vaccine strains [16–22], rendering the vaccine less effective in protecting against street RABV variants [19,23].

RABV has high mutation rate due to the low fidelity of viral polymerase [24], resulting in a diverse pool of RABV [25]. While the non-synonymous/synonymous ratio is relatively low, the antigenic relationship between epidemic RABVs and current vaccine strains must be constantly monitored, given that rabies is a fatal disease. Here, sequences of RABV G protein of all street isolates and vaccine strains were retrieved from public domain and analysed. A total of 99 mutations were identified and subsequently incorporated into pseudoviruses for high throughput analyses of their antigenicity using well-characterized mAbs and vaccine-induced antibodies. We found marked variations in their reactions to the antibodies known to target the protective epitopes.

## Materials and methods

### MAbs and vaccines

MAb CTB011 and CTB012 were components of SYN023 [26], a therapeutic regimen obtained from Synermore Biologics Co., Ltd (Suzhou, China). CTB011 targets residues near antigenic site III while CTB012 binds to the highly discontinuous conserved residues. MAb NM57S (anti-antigenic site I) and NC08 (anti-antigenic site II) were components of an antibody regimen in phase III clinical trial, obtained from New Drug R&D Center (North China Pharmaceutical Corporation, Shijiazhuang, China). RVAB3 and RVAB5 were antibodies against antigenic site II. A commercial human rabies immunoglobulin (HRIG) was obtained from Tonrol Bio-Pharmaceutical Co., Ltd (Hefei, China). The 37.0 IU/ml national standard for anti-rabies immunoglobulin was established and calibrated by National Institutes for Food and Drug Control (NIFDC); it was used to calculate the neutralizing antibody titre of serum samples from human vaccines.

Commercial vaccines comprised of vaccine strains Flury-LEP, aGV, CTN-1V, PM, and PV-2061 have been determined as ≥2.5IU/dose for each strain.

### Animals and immunizations

All animal studies were approved by the Institutional Animal Care and Use Committee of NIFDC. Female Hartley guinea pigs (*n* = 6/group) were immunized intramuscularly with 0.2 mL of each reconstituted rabies vaccine (1.0 mL) at weeks 0 and 1. Serum samples were obtained 1 week after the last immunization. Serum samples were stored at −70°C; they were heat-inactivated at 56°C for 1 h before use.

### Mutagenesis and pseudovirus-based neutralization assays

The CVS-N2c glycoprotein expressing plasmids psCMV.CVS-N2c and backbone plasmid pSG3ΔEnv.sCMV.fluc were constructed as described previously [27]; AA substitution was performed using site-directed mutagenesis on plasmid psCMV.CVS-N2c [28], while pseudovirus preparation, titration, and neutralization assays were performed as described previously [27]. To assess reactivities of mutants to vaccine-induced antibodies, pooled sera from six animals immunized with the same vaccine were used for initial evaluation; once a mutant virus showed >4-fold ID_50_ change compared to wild-type CVS-N2c, each antiserum of this vaccine group was further evaluated.

### RABV sequences analysis

All full-length G amino acid sequences (524 AA) of rabies street isolates and seven vaccines were downloaded from GenBank. Only isolates with full background information were considered. The MEGA6 [29] was used to calculate the identity distance matrix of the extracellular domain between vaccine strains and street isolates. For each vaccine, a simple linear regression model (R lm function) was used to analyse the AA sequence similarity between the street isolates and vaccine. The Genbank ID of human rabies vaccine strains are as follows: PV (P08667.1), PM (CAI43218.1), Flury-LEP (ADD84785.1), aGV (ADM32132.1), CTN-1V (ACR39382.1), Vnukovo-32 (CAA50713.1), and PV-2061 (AEV43289.1).

### Statistical analysis

A simple linear regression model (R lm function) was used to analyse the AA sequence similarity between the street isolates and vaccines with time. A *p*-value of less than 0.05 is considered significant. In the vaccine potency and neutralization assay, statistical significance was determined using SPSS 19.0. Differences in five vaccine potency tests were analysed using One-way analysis of variance test with Tukey’s multiple comparison, while neutralization assay was analysed using One-way analysis of variance test with Kruskal–Wallis.

## Results

### Evolution of glycoprotein sequences of street RABVs and its relationship with human vaccine strains

**Figure 1.**
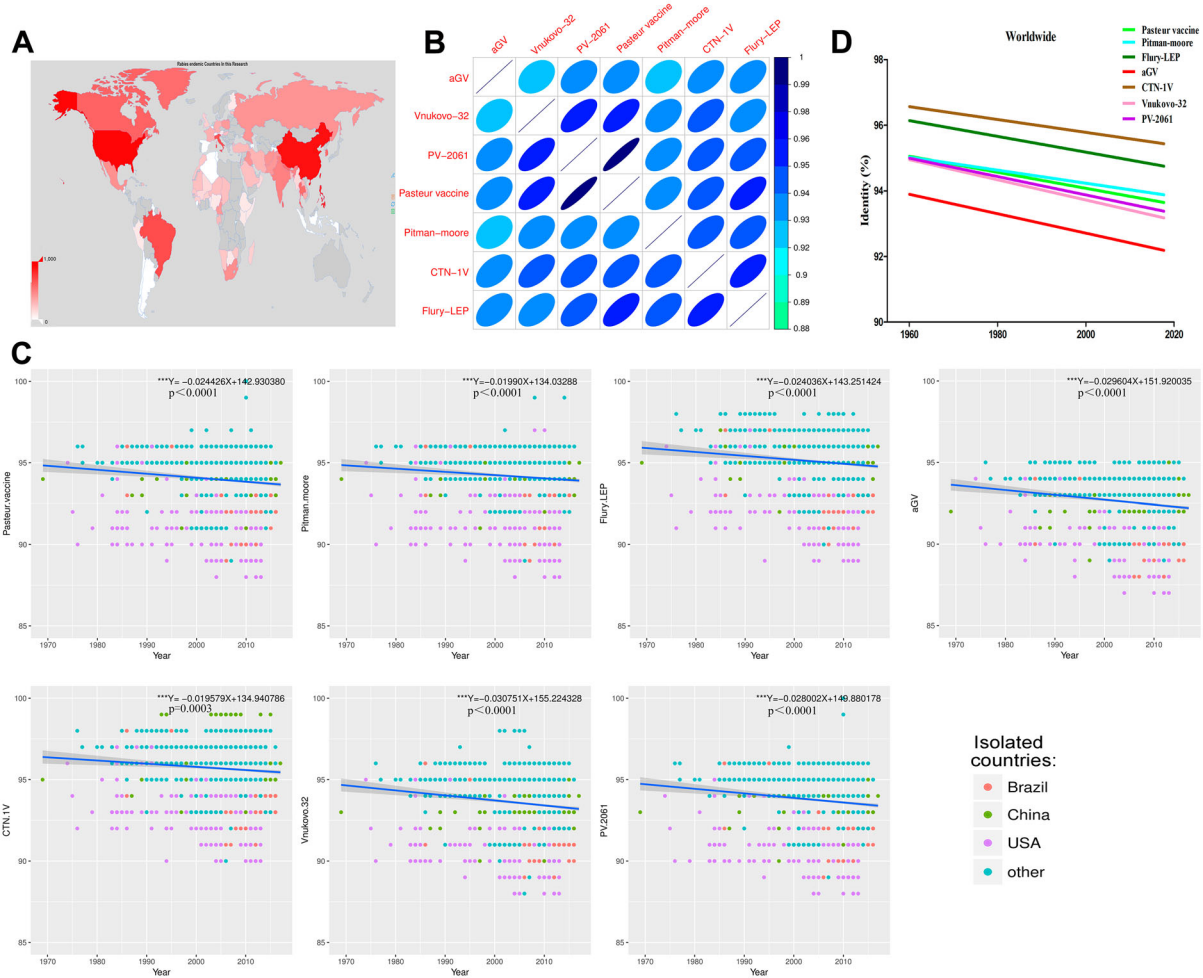
Sequence analysis of the surface glycoprotein of RABV Street isolates. (A) Geographical distribution of collected RABV Street isolates.(B) Heat map of seven RABV vaccine strains based on sequence similarity.(C) Sequence variation of RABV Street isolates compared to vaccines with time.(D) Sequence variation of RABV Street isolates worldwide compared to vaccines. If *p*-value of the slope of the regression equation was below 0.05, it was considered significant.“*”*p* < 0.05, “**” *p* < 0.01, “***” *p* < 0.001.

2890 AA sequences of RABV G protein were obtained worldwide from Genbank, with most isolates derived from Asia, Africa, South America, and North America. Notably, China, USA, and Brazil are the main sources of RABV isolates (Figure 1(A)). As shown in Figure 1(B), the seven vaccine strains had high degree of similarity (over 92%), where PV and PV-2061 shared the highest identity (99%). Globally, the street isolates from 1960 have a trend of decreasing similarity to all seven vaccine strains (*p* < 0.001, Figure 1(C)), with the most remarkable found with the vaccine strains aGV and Vnukovo-32 (largest slope, Figure 1(D)). The vaccine strains CTN-1V and Flury-LEP shared the highest degree of identities to all street RABVs, whereas the aGV had the lowest (Figure 1(D)). The decreasing trend between street strains and vaccine strains was observed in all geographic locations, with isolates from Brazil and South America showing the sharpest drop in sequence similarities (Supplementary Figure 1).

### Amino acid selection for mutagenesis

We next aligned 2890 RABV G proteins and identified AA at each position in the extracellular domain. A total of 99 naturally occurring mutations were selected and incorporated into pseudoviruses using CVS-N2c as backbone for high throughput neutralizing assays. Specifically, there are six antigenic sites on RABV G protein including sites I, II (IIa & IIb), III, IV, G5, and minor site a [30–35]; the AA in the six antigenic sites was relatively conservative. Finally, 65 mutations were introduced individually in the antigenic sites (Supplementary Table 1). The other 34 mutations with frequencies of over 10% were introduced into region outside the aforementioned antigenic sites (Supplementary Table 2).

### Effect of mutations on neutralization by mAbs

Of all mutants, C35S, S39P/F, R199G, N204S/G, C228S, V332F, I338F, and G343W showed significant reduction of viral infectivity or pseudovirus formation. Interestingly, despite the low infectivity or pseudovirus formation for mutants S39P/F, R199G, V332F, and I338F, alternative mutations on the same positions, i.e. S39T, R199K, V332I, I338T/V, showed comparable viral infectivity as CVS-N2c. All the mutants, except those showing low infectivity or pseudovirus formation (ten mutants), were characterized for their reactivities to six well-characterized neutralizing mAbs.

**Figure 2.**
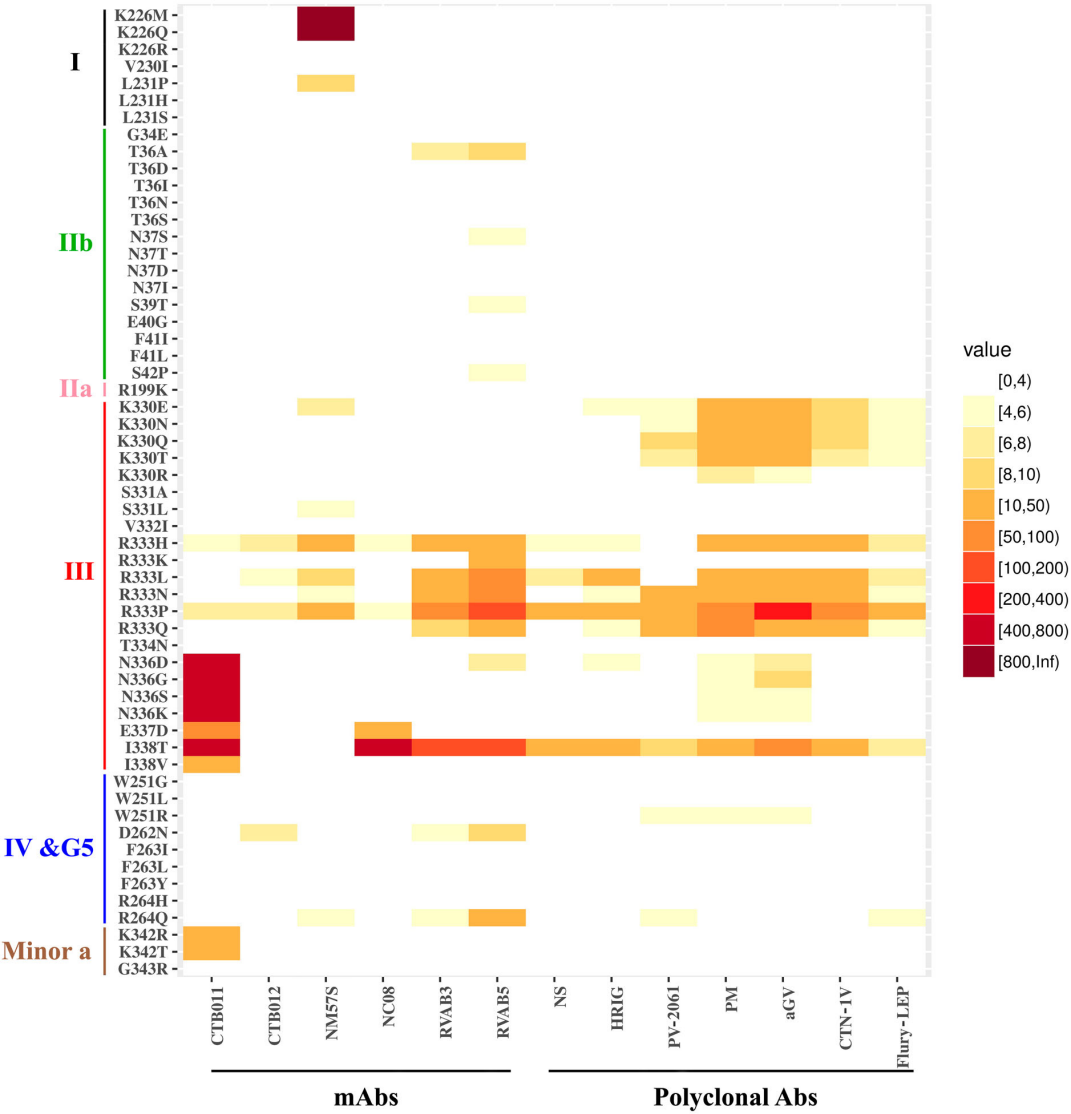
Sensitivity of G protein mutants to neutralization by mAbs and polyclonal Abs. The level of decreased of sensitivity to Ab-mediated neutralization is present by different colours, i.e. under 4-fold change in white. NS in polyclonal Abs panel denotes the national anti-rabies standard. Data are from three independent experiments.

We found mutations in antigenic site III resulted in marked resistance to those neutralizing mAbs. Mutations at R333, specifically R333P and R333H, have reduced reactivity to all mAbs, while R333P was resistant to mAb RVAB5 as demonstrated by the highest IC_50_ (40 ng/mL), a 53-fold reduction in susceptibility to neutralization by RVAB3, 14-fold to mAb NM57S, ∼8-fold to CTB011, ∼5-fold to both NC08 and CTB012. Moreover, R333K/Q mutation has less impact compared to R333P (Figure 2).

Other mutations on antigenic site III, including N336D/G/S/K, E337D, and I338T/V, demonstrated significant resistance to one neutralizing mAb CTB011 (Figure 2). The I338T/V is an interesting pair of mutants. Specifically, while I338T had a much higher reduction (over 588-fold, resistant to highest IC_50_) of sensitivity to mAb CTB011 than I338 V (∼11-fold), it is also over 100-fold more resistant other three mAbs (NC08, RVAB3, and RVAB5) (Figure 2).

It is of note that within antigenic site I, only mutations of K226M/Q led to the virus escape from NM57S as revealed by the highest IC_50_. However, K226R mutation, which maintains the size and charge property, had no effect on NM57S mediated neutralization (Figure 2).

In antigenic sites IIb, IV, G5, minor site a, and other regions outside the antigenic sites, only a few mutants showed a modest resistance to some mAbs (Figure 2 & Supplementary Table 3).

### Effect of mutations on neutralization by vaccine-induced antibodies

**Figure 3.**
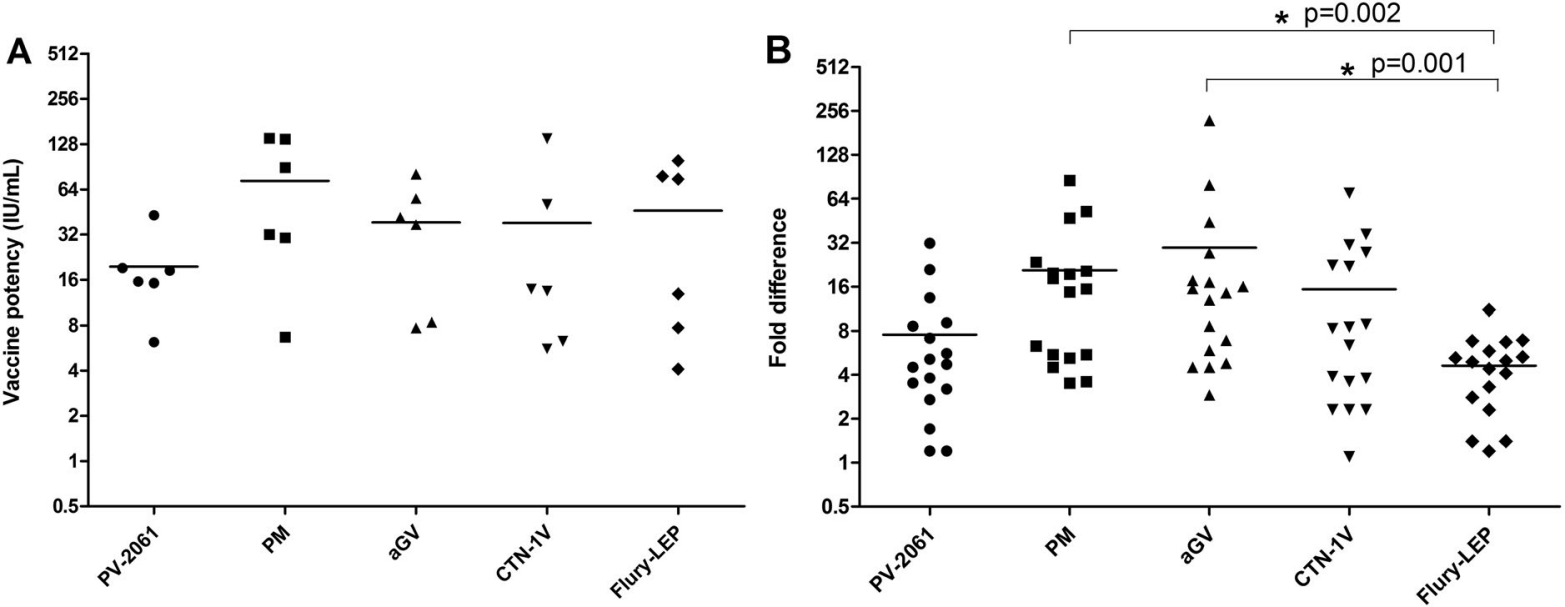
Comparison of vaccine potency and ability to neutralize viral mutants by different vaccine strains.(A) Five commercial rabies vaccines with different vaccine strains were used, i.e. Flury-LEP, aGV, CTN-1 V, Pitman-Moore (PM) and PV-2061 vaccine, to immune guinea pigs (*n* = 6/group) intramuscularly with 0.2 mL of each rabies vaccine at weeks 0 and 1. The potency was measured by CVS-N2c pseudovirus-based neutralization assay, with calculation by National antibody reference standards (37.0 IU/mL). *P* values were calculated by One-way analysis of variance (Tukey’s multiple comparison Test, SPSS 19·0). (B) Mutations, W251R, R264Q, K330Q/R/N/E/T, R333L/P/Q/N/H, N336D/G/S/K, and I338 T, with >4-fold increase in resisting to vaccine-induced antibodies, were subjected to the analyses on tolerance of vaccines to these mutations. *P* values were calculated by using One-way analysis of variance (Kruskal-Wallis test, SPSS 19.0). The difference between vaccines is considered significant when the *p*-value is < 0.05, as indicated with an asterisk.

All five vaccines induced similarly high levels (>4 IU/mL) of neutralizing antibodies (Figure 3(A)). The pooled antisera were tested for their neutralizing activities against all mutant pseudoviruses. Consistent with the mAb assays, mutations in antigenic site III resulted in the strongest resistance to the polyclonal antibodies in the pooled antisera. Specifically, mutation at R333 except R333K significantly reduced the neutralization susceptibility, with R333P a 13-fold ID_50_reduction to the Ab standard, a 27-fold reduction to HRIG, and a 11−220-fold reduction to the pooled antisera (Figure 2). Other R333 mutations had less effect on neutralization sensitivity than R333P, with no altered reactions observed with R333K. The I338T had a 10-fold reduction to the Ab standard, 16-fold reduction to HRIG and a 7−80-fold reduction to the pooled antisera. However, I338V had no effect on the neutralization phenotype. K330 mutations at the tip of antigenic site III showed a 4−20-fold reduction to vaccine-induced antibodies. N336 mutations had a 4−9-fold reduction to PM and aGV vaccine-induced antibodies.

Furthermore, no significant alterations to antibody neutralization were associated with mutations in the antigenic site I, II, and minor site a since the reduced sensitivity is less than 4-fold. In antigenic site IV and G5, only W251R and R264Q showed about 4-fold reduction to some vaccine-induced antibodies. Mutants in other regions of the extracellular domain outside the antigenic sites also showed less than 4-fold in ID_50_.

**Figure 4.**
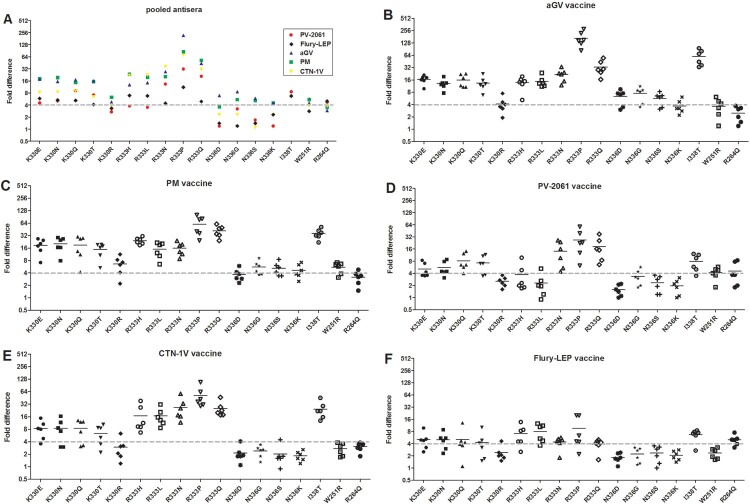
Effects of viral mutations on neutralizations by vaccine-induced polyclonal antisera. Mutations with >4-fold ID_50_ compared to wild-type CVS-N2c were shown. (A) Neutralization by pooled antisera from 6 animals immunized with the same vaccine. Serum sample from testing group was further evaluated as (B) for aGV vaccine, (C) PM vaccine, (D) PV-2061 vaccine, (E) CTN-1 V vaccine and (F) Flury-LEP vaccine. Fold difference was calculated by dividing the mean ID_50_ (WT) by mean ID_50_ (mutant). Data are from three independent experiments.

For mutants with over 4-fold change in ID_50_ in their reactions to the pooled antisera (mutation W251R, R264Q, K330Q/R/N/E/T, R333L/P/Q/N/H, N336D/G/S/K, and I338T), serum sample from each vaccine group was further evaluated. As shown in Figure 4, these mutants showed similar levels of resistance to neutralization by the pooled antisera.

We next investigated how strain-specific vaccine-induced antibodies would neutralize the mutants with over 4-fold reduction in neutralization assay. These mutants, W251R, R264Q, K330Q/R/N/E/T, R333L/P/Q/N/H, N336D/G/S/K, and I338T, were analysed in neutralization assay using strain-specific antisera induced by a different vaccine. As shown in Figure 3, although the five commercial vaccine-induced similar levels of neutralizing antibody titre (Figure 3(A)), they varied in their neutralizing activities against RABV mutants. Specifically, the ID_50_ of PV-2061 and Flury-LEP to mutants were only reduced by approximately 4-fold, while larger reductions in ID_50_ values were observed with PM, aGV, and CTN-1V (Figure 3(B)), with ID_50_ of aGV and PM vaccines being greater than Flury-LEP (*p* < 0.05, Figure 3(B)).

### Geographic distribution of street mutants with increased resistance to vaccine-induced antibodies

**Figure 5.**
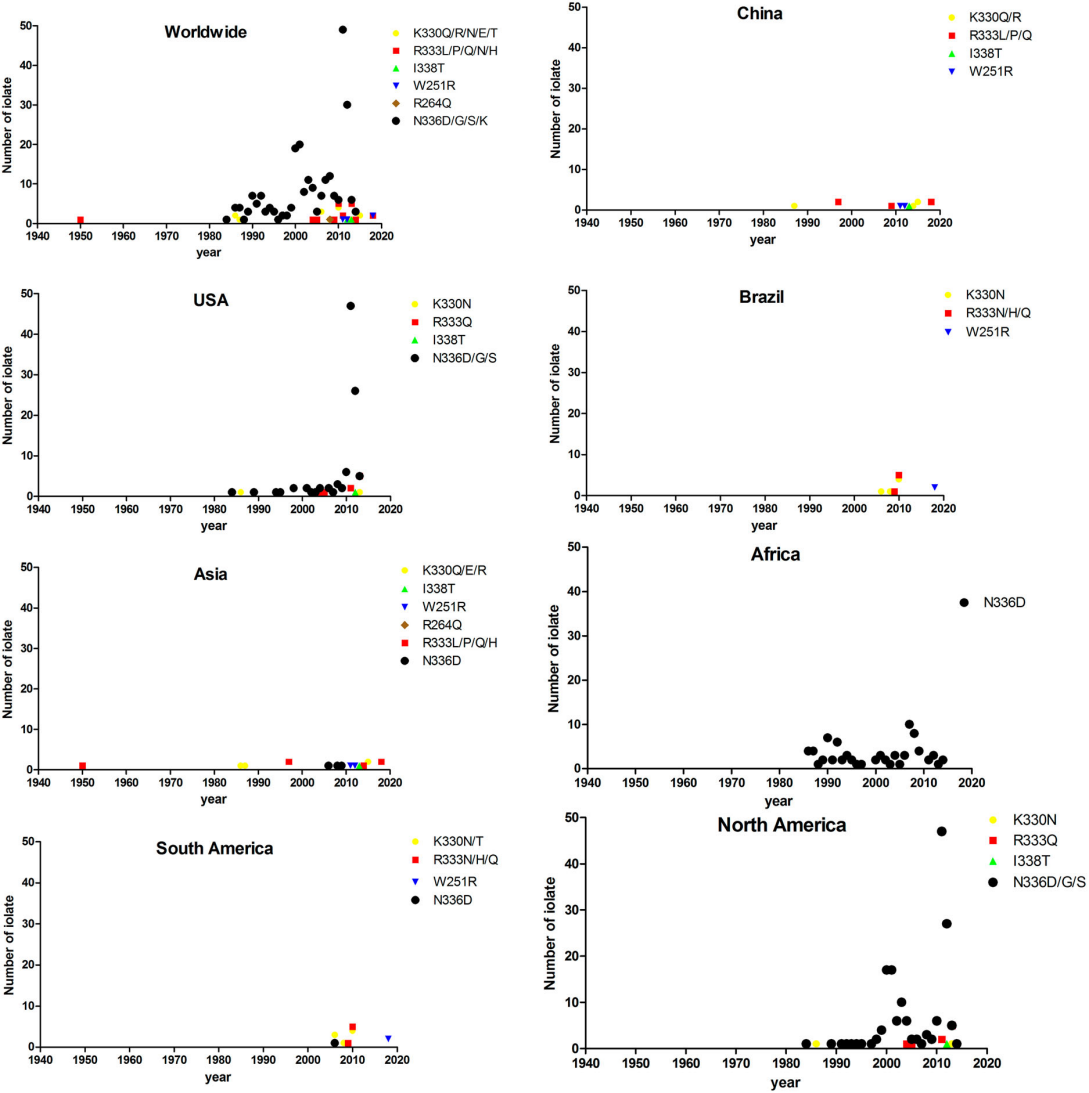
The identification time and geographical distribution of street RABV mutants resistant to vaccine sera neutralization.

We analysed the geographic distribution, isolation time, and hosts for mutants resistant to vaccine-induced antibodies. Specifically, W251R, R264Q, K330Q/R/N/E/T, R333L/P/Q/N/H, N336D/G/S/K, and I338T were analysed. Globally, these street virus strains were isolated around 2010 and their number increased with time (Figure 5). The percentage of the variants among all strains is as follows: K330Q/R/N/E/T is 0.5%, R333L/P/Q/N/H is 0.71%, N336D/G/S/K is 8.68%, W251R is 0.14%, R264Q is 0.03%, and I338T is 0.07% (Supplementary Table 1).

Notably, these variants showed a wide range of hosts including dogs, cats, bats, Homo sapiens, and other mammals. Furthermore, they have predominantly been isolated from Asia, Africa, South America, and North America. China is the major source of isolates in Asia, the USA in North America, and Brazil in South America, whereas in Africa, no country was found to be the main source of mutants. Notably, some variants were found in certain geographic locations. Specifically, the R333P was isolated only in China, the I338T in China and USA, W251R in China and Brazil, and the R264Q in India.

It is also noted that 330/333/336 variants were isolated from different geographic regions, and the exact AA at each position was different among these regions. Specifically, K330Q/E/R was isolated from Asia and K330N/T in South America and North America. Notably, the number of N336 variants (N336D/G/S) had increased since 2010 in the USA/North American.

**Figure 6.**
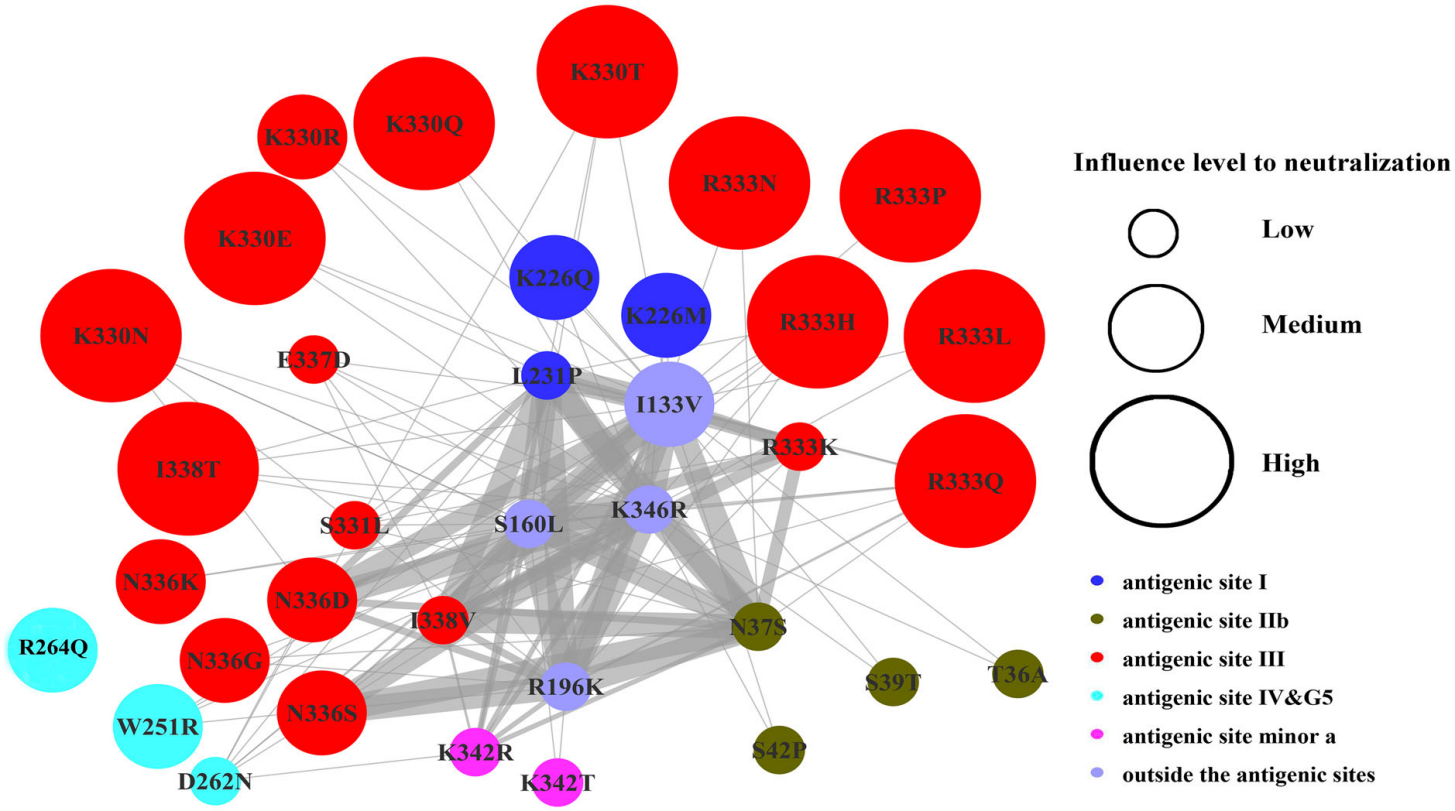
Coexistence of mutations. Node size corresponds to the impact on neutralization (Low, Medium and High). Low: mutations which showed a 4−10-fold reduction to 1−3 mAbs, or 4−10-fold to one polyclonal Abs; Medium: mutations resistant to 1−2 mAbs, or with 4−10-fold reduction to 2−3 polyclonal Abs; High: mutations with over 4-fold reduction to at least four mAbs, or at least 4-fold reduction to at least four polyclonal Abs. The thickness of the connecting line between nodes indicates the frequency of mutation coexistence, with the thicker lines representing higher frequency. Node colour depicts different antigenic sites or regions in RABV glycoprotein.

To examine whether these AA substitutions coexisted with each other, we analysed their coexistence frequency. As shown in Figure 6, while mutations W251R, R264Q, K330Q/R/N/E/T, R333L/P/Q/N/H, N336D/G/S/K, and I338T demonstrated the greatest resistance to vaccine-induced antibodies, they were not found to coexist with each other; nevertheless, co-existence was found inmutants with 4–10 fold increase in resistance to mAbs. Of these mutants, coexistence was more frequent in N37S, I133V, S160L, R196K, R333K, I338V, and K346R.

## Discussion

Genetic analyses are widely used to predict cross-protection induced by rabies vaccine; however, such analysis is of limited value to predict the effects of AA substitutions on antigenicity. Indeed, over 30% of the antigenic variation could not be predicted by genetic distance alone [36]. Here, we systematically analysed RABV glycoprotein sequences in the public domain and incorporated the mutations into a pseudoviral system for high throughput analyses of their sensitivity to well-characterized mAbs and vaccine-induced antisera.

We found a trend of decreasing sequence similarity between RABV street isolates and vaccine strains; moreover, the slope and similarity between street isolates and each vaccine strain were found to differ. We also observed some mutants, particularly those with mutations on the antigenic site III, are much more resistant to vaccine-induced antisera. Since both B- and T-cell mediated immune response can control virus infection, whether these neutralization resistant viruses could be protected *in vivo*, need to be elucidated by further performing the challenge studies in animal models following different rabies vaccinations or post-exposure prophylaxis (PEP).

These mutants were isolated around 2010 and increased in number onward. At this time, the prevalence of significant neutralization resistant variants was very low, specifically, R333 mutants only constituted 0.71% of all RABV strains. However, the most commonly isolated variants such as N336 mutants, did not require significantly increased concentration of neutralization dose. Although the proportion of these variants in all strains were low (Supplementary Table 1), these mutants still have the potential to evolve and become the dominant in the future. To make sure the current vaccines and antibodies for PEP are able to protect from RABV infections, isolation and sequence determination of resistant viruses are very important. It should be noted that although the number of mutant virus isolates is fewer in Asia and South America, the most resistant mutants were also isolated in these regions, thus, the surveillance in these regions still needs to be strengthened.

Mutations in antigenic site III resulted in the strongest resistance to the tested mAbs and antisera in our study, especially the R333 and I338T mutations. R333 in antigenic site III is known to be involved in the viral pathogenicity [32], as demonstrated by reduced viral pathogenicity in mutant with substitution at this position. Here, we showed that R333 mutations became significantly resistant. Importantly, we found AA at position 333 is highly conserved in lyssavirus genus albeit the exact amino acid may differ in different phylogroups. Specifically, R333 is found in the majority of phylogroup I whereas R333 was not found in phylogroups II and III. This might explain that the current rabies vaccines confer better protection against phylogroup I lyssavirus [6–9]. The I338T mutation has also demonstrated increased resistance to neutralizing antibody, a mechanism yet to be understood. It is likely, however, the mutation may have introduced N-linked glycosylation at N336 (N336-E337-T338) hindering the interaction of the viral surface with neutralizing antibodies.

In this study, while the individual mAb showed differences in neutralizing mutants, cocktails of those (particularly CTB011/012; NM57S/NC08) showed broad coverage, and are generally able to neutralize all RABV mutants. This suggested that the combination of mAbs that bind to different antigenic sites on the RABV G protein will significantly reduce the risk of PEP failure.

The WHO has set a goal to eliminate fatal cases of human rabies by 2030. Although the street mutants resistant to neutralizing antibodies constitute less than 10% of all RABV strains at this time, there appears to be a trend that these mutants have been increasing in recent years. Given the wide range of hosts and geographic locations including the countries previously reported to be free of canine rabies, close monitoring of emerging mutants should be conducted as they may potentially render the current rabies vaccine less effective.

## Supplementary Material

Supplemental Material
