# Supplementary material for: Antigenic variations of recent street rabies virus
Source: Emerg Microbes Infect. 2019 Nov 4;8(1):1584–92. doi: 10.1080/22221751.2019.1683436 (PMC6844422; doi:10.1080/22221751.2019.1683436)
Supplement: Supplemental Material [file TEMI_A_1683436_SM9674.docx]

**Supplementary Figure 1.** Analysis of the similarity variation tendency of RABV street isolates in different regions.


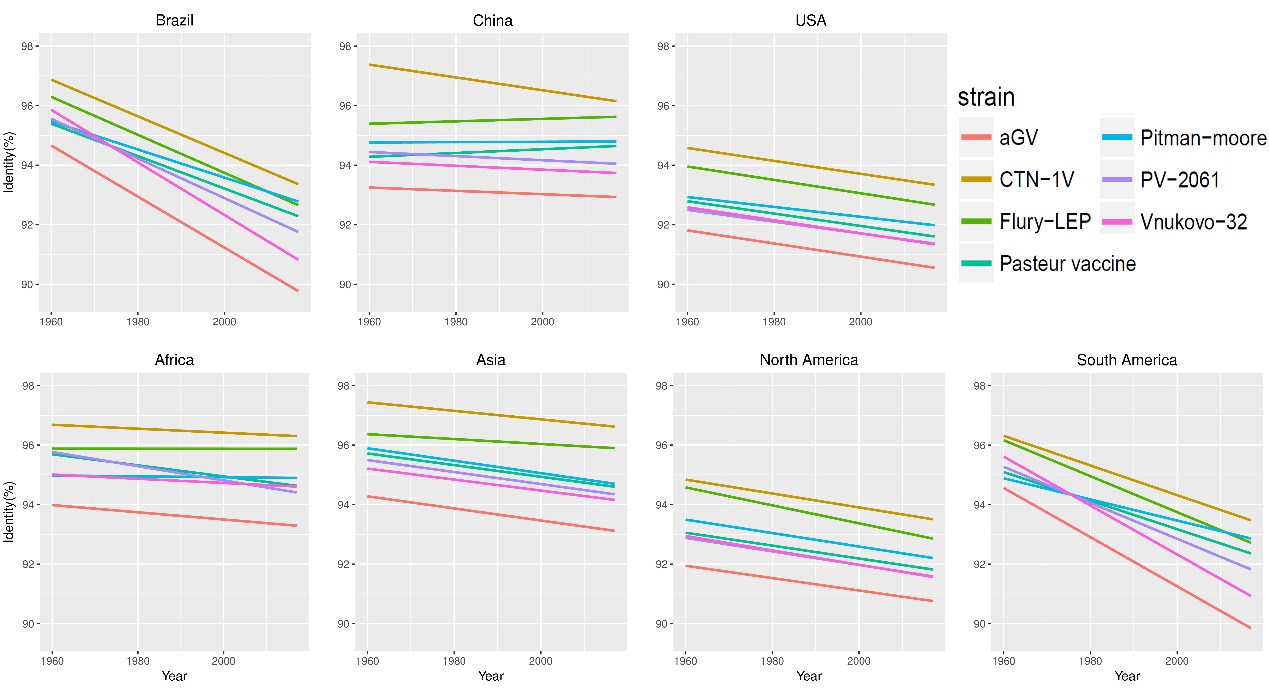


**Supplementary Table 1. Amino Acid occurrence frequency within the antigenic sites of the 2890 RABV isolates.**

| **Antigenic site** | **Position** | **Amino Acid distribution (%)** |
| --- | --- | --- |
| **I** | 226 | ***K(99***·***69)***, M(0·14), Q(0·03), R(0·14) |
|  | 227 | ***L (100)*** |
|  | 228 | ***C (99***·***97)***, S(0·03) |
|  | 229 | ***G (100)*** |
|  | 230 | I(0·14), ***V (99***·***86)*** |
|  | 231 | H(0·03), ***L (72***·***28)***, P(12·94), S(14·74) |
| **IIa** | 198 | ***K (100)*** |
|  | 199 | G(0·03), K(7·61), ***R (92***·***35)*** |
|  | 200 | ***A (100)*** |
| **IIb** | 34 | E(0·10), ***G (99***·***90)*** |
|  | 35 | ***C (99***·***97)***, S(0·03) |
|  | 36 | A(0·14), D(1·07), I(0·03), N(15·36), S(0·17),***T (83***·***22)*** |
|  | 37 | D(0·03), I(0·03),***N (76***·***40)***, S(22·87), T(0·66) |
|  | 38 | ***L (100)*** |
|  | 39 | F(0·03), P(0·03), ***S (99***·***90)***, T(0·03) |
|  | 40 | ***E (0***·***10)***, G(99·90) |
|  | 41 | ***F (99***·***72)***, I(0·21), L(0·07) |
|  | 42 | P(0·03), ***S (99***·***97)*** |
| **III** | 330 | E(0·03), ***K (99***·***48)***, N(0·31), Q(0·03), R(0·10), T(0·03) |
|  | 331 | A(0·03), L(0·03), ***S (99***·***93)*** |
|  | 332 | F(0·07), I(19·93), ***V (80***·***0)*** |
|  | 333 | H(0·10), K(1·90), L(0·03), N(0·10), P(0·03), Q(0·45), ***R (97***·***37)*** |
|  | 334 | N(0·03), ***T (99***·***97)*** |
|  | 335 | ***W (100)*** |
|  | 336 | D(3·98), G(0·10), K(0·03), ***N (91***·***31)***, S(4·57) |
|  | 337 | D(0·21), ***E (99***·***79)*** |
|  | 338 | F(0·03), ***I (95***·***09)***, T(0·07), V(4·81) |
| **IV&G5** | 251 | L(0·03), G(0·10), R(0·14), ***W (99***·***72)*** |
|  | 261 | ***H (100)*** |
|  | 262 | ***D (99***·***79)***, N(0·21) |
|  | 263 | ***F (89***·***24)***, I(0·07), L(10·66), Y(0·03) |
|  | 264 | H(45·74), Q(0·03), ***R (54***·***22)*** |
| **Minor a** | 342 | ***K (98***·***41)***, R(1·56), T(0·03) |
|  | 343 | ***G (99***·***93)***, R(0·03), W(0·03) |
| **AA in CVS-N2c are shown as bold italic, and the bold font AA was mutated to unbolded AA at each position and the 100% conserved AA was excluded from the mutagenesis.** | | |

**Supplementary Table 2. List of mutations in another region of the extracellular domain outside the antigenic sites.**

| **Position** | **Mutation** |
| --- | --- |
| 90 | T90M/I |
| 96 | A96S |
| 102 | M102L |
| 113 | H113Q |
| 133 | I133V |
| 153 | V153I |
| 156 | G156S |
| 160 | S160L |
| 164 | V164I |
| 168 | Y168C |
| 179 | M179L |
| 182 | N182K/E |
| 183 | P183A |
| 184 | R184G |
| 194 | N194T |
| 196 | R196K |
| 204 | N204S/G |
| 205 | K205R |
| 241 | V241A |
| 243 | M243I |
| 249 | T249I |
| 253 | P253S |
| 278 | K278R |
| 289 | S289T |
| 346 | K346R |
| 370 | H370N |
| 382 | Q382H |
| 408 | E408D |
| 409 | G409D |
| 427 | I427V |
| 436 | N436S |

**Supplementary Table 3. Fold change of neutralization susceptibility of G protein mutants to antibodies.**

|  | **mAbs** | | | | | | ***polyclonal Abs and vaccine sera*** | | | | | | |
| --- | --- | --- | --- | --- | --- | --- | --- | --- | --- | --- | --- | --- | --- |
| **Mutants** | **CTB011** | **CTB012** | **NM57S** | **NC08** | **RVAB3** | **RVAB5** | ***Ab standard*** | ***HRIG*** | ***PV-2061*** | ***PM*** | ***aGV*** | ***CTN-1V*** | ***Flury-LEP*** |
| **CVS-N2c** | 1 | 1 | 1 | 1 | 1 | 1 | 1 | 1 | 1 | 1 | 1 | 1 | 1 |
| **T90M** | 1.0 | 1.3 | 1.1 | 1.3 | 1.5 | 1.5 | 1.6 | 1.2 | 1.3 | 1.6 | 2.0 | 1.5 | 1.2 |
| **T90I** | 0.9 | 1.4 | 1.3 | 1.0 | 1.3 | 1.8 | 1.8 | 1.4 | 1.6 | 2.1 | 1.7 | 1.2 | 1.2 |
| **A96S** | 1.5 | 2.2 | 2.1 | 1.8 | 2.1 | 2.9 | 1.9 | 2.0 | 1.8 | 2.2 | 1.9 | 2.3 | 2.0 |
| **M102L** | 0.9 | 1.9 | 1.3 | 1.5 | 1.7 | 1.6 | 1.2 | 1.7 | 1.4 | 1.6 | 2.0 | 1.4 | 1.4 |
| **H113Q** | 0.9 | 2.0 | 1.7 | 1.8 | 1.6 | 1.6 | 2.0 | 1.0 | 1.9 | 2.0 | 2.3 | 2.3 | 1.6 |
| **I133V** | 2.2 | 2.9 | 3.2 | 2.4 | 4.2 | 5.5 | 4.6 | 4.1 | 1.5 | 1.5 | 1.5 | 3.5 | 2.4 |
| **V153I** | 1.5 | 2.6 | 1.9 | 2.4 | 1.8 | 2.9 | 2.1 | 1.6 | 1.8 | 2.3 | 2.2 | 3.6 | 1.4 |
| **G156S** | 1.5 | 2.2 | 1.9 | 1.5 | 1.4 | 1.7 | 1.6 | 1.6 | 1.3 | 1.4 | 1.5 | 1.9 | 1.2 |
| **S160L** | 1.5 | 2.3 | 0.9 | 1.2 | 2.4 | 3.2 | 5.3 | 3.5 | 1.0 | 2.4 | 1.2 | 2.6 | 0.9 |
| **V164I** | 1.6 | 2.5 | 2.4 | 2.1 | 1.4 | 1.8 | 2.1 | 2.0 | 1.9 | 2.0 | 1.6 | 2.6 | 1.5 |
| **Y168C** | 1.2 | 1.5 | 1.6 | 1.2 | 1.2 | 2.0 | 1.6 | 1.6 | 1.3 | 1.5 | 1.4 | 1.9 | 1.1 |
| **M179L** | 1.6 | 2.3 | 1.4 | 1.2 | 1.3 | 1.3 | 2.2 | 2.1 | 1.2 | 1.3 | 1.1 | 2.0 | 1.2 |
| **N182K** | 1.7 | 1.1 | 0.3 | 0.5 | 2.3 | 2.9 | 2.7 | 3.2 | 1.3 | 1.8 | 1.5 | 3.0 | 1.7 |
| **N182E** | 0.8 | 1.3 | 0.8 | 0.8 | 0.7 | 0.8 | 1.8 | 1.2 | 0.7 | 1.0 | 0.6 | 2.0 | 0.6 |
| **P183A** | 1.8 | 2.0 | 1.6 | 1.5 | 1.2 | 1.7 | 2.5 | 2.5 | 1.5 | 1.9 | 1.6 | 2.6 | 1.2 |
| **R184G** | 0.8 | 0.8 | 0.5 | 0.9 | 0.5 | 0.7 | 0.6 | 0.5 | 1.0 | 1.0 | 0.7 | 1.1 | 0.5 |
| **N194T** | 0.9 | 1.2 | 1.3 | 1.5 | 1.3 | 1.2 | 1.2 | 1.4 | 1.2 | 1.3 | 1.3 | 1.4 | 1.1 |
| **R196K** | 2.0 | 2.8 | 1.8 | 2.1 | 4.8 | 5.6 | 2.9 | 2.5 | 2.2 | 3.5 | 2.9 | 1.7 | 2.3 |
| **K205R** | 0.7 | 1.2 | 0.6 | 1.0 | 0.9 | 1.0 | 1.1 | 0.8 | 0.5 | 0.8 | 0.5 | 1.6 | 0.3 |
| **V241A** | 1.0 | 1.3 | 1.3 | 1.5 | 1.3 | 1.3 | 1.2 | 1.4 | 1.0 | 1.5 | 1.4 | 1.4 | 0.7 |
| **M243I** | 1.1 | 1.8 | 1.2 | 1.6 | 1.0 | 1.7 | 1.5 | 1.2 | 2.2 | 2.2 | 2.6 | 2.3 | 1.8 |
| **T249I** | 1.0 | 1.4 | 1.1 | 1.0 | 0.9 | 1.0 | 1.2 | 1.3 | 0.9 | 1.1 | 1.3 | 1.4 | 0.7 |
| **P253S** | 0.9 | 0.8 | 0.8 | 0.9 | 0.8 | 0.8 | 1.2 | 1.0 | 0.7 | 1.3 | 0.8 | 1.6 | 0.3 |
| **K278R** | 0.8 | 1.0 | 0.6 | 0.9 | 0.7 | 0.8 | 1.6 | 1.7 | 0.7 | 1.4 | 0.8 | 1.8 | 0.6 |
| **S289T** | 1.7 | 3.3 | 1.6 | 2.1 | 2.9 | 3.6 | 2.5 | 2.3 | 0.9 | 1.8 | 1.3 | 2.4 | 0.5 |
| **K346R** | 2.1 | 2.2 | 2.1 | 1.3 | 2.3 | 2.0 | 2.6 | 1.8 | 1.3 | 3.1 | 1.2 | 1.8 | 1.4 |
| **H370N** | 1.4 | 2.2 | 1.9 | 1.6 | 1.8 | 2.7 | 2.5 | 2.3 | 1.5 | 1.4 | 1.5 | 2.3 | 1.8 |
| **Q382H** | 0.9 | 1.3 | 0.9 | 0.9 | 1.0 | 1.1 | 1.1 | 1.2 | 1.0 | 1.4 | 1.5 | 1.5 | 1.1 |
| **E408D** | 1.1 | 1.4 | 1.5 | 1.4 | 1.2 | 1.2 | 1.6 | 2.0 | 0.9 | 1.2 | 1.1 | 1.5 | 0.8 |
| **G409D** | 2.3 | 2.5 | 1.9 | 1.9 | 1.8 | 2.7 | 3.6 | 3.4 | 1.7 | 1.9 | 1.7 | 2.7 | 1.8 |
| **I427V** | 0.9 | 1.4 | 1.3 | 1.4 | 1.5 | 1.4 | 1.4 | 1.2 | 1.1 | 1.2 | 1.5 | 1.5 | 0.7 |
| **N436S** | 1.1 | 1.5 | 1.2 | 1.2 | 1.3 | 1.8 | 1.8 | 1.5 | 1.9 | 1.3 | 1.4 | 2.1 | 1.9 |
| **polyclonal Abs and vaccine sera are shown as italic** | | | | | | | | | | | | | |
